# NMR-based metabolomics Reveals Alterations of Electro-acupuncture Stimulations on Chronic Atrophic Gastritis Rats

**DOI:** 10.1038/srep45580

**Published:** 2017-03-30

**Authors:** Jingjing Xu, Xujuan Zheng, Kian-Kai Cheng, Xiaorong Chang, Guiping Shen, Mi Liu, Yadong Wang, Jiacheng Shen, Yuan Zhang, Qida He, Jiyang Dong, Zongbao Yang

**Affiliations:** 1 Department of Electronic Science, and Department of Traditional Chinese Medicine, Xiamen University, Xiamen 361005, China; 2 Department of Bioprocess & Polymer Engineering, Innovative Centre in Agritechnology, University Teknologi Malaysia, Johor 81310, Malaysia; 3 College of Acupuncture and Moxibustion, Hunan University of Chinese Medicine, Changsha 410007, China

## Abstract

Chronic atrophic gastritis (CAG) is a common gastrointestinal disease which has been considered as precancerous lesions of gastric carcinoma. Previously, electro-acupuncture stimulation has been shown to be effective in ameliorating symptoms of CAG. However the underlying mechanism of this beneficial treatment is yet to be established. In the present study, an integrated histopathological examination along with molecular biological assay, as well as ^1^H NMR analysis of multiple biological samples (urine, serum, stomach, cortex and medulla) were employed to systematically assess the pathology of CAG and therapeutic effect of electro-acupuncture stimulation at *Sibai* (ST 2), *Liangmen* (ST 21), and *Zusanli* (ST 36) acupoints located in the stomach meridian using a rat model of CAG. The current results showed that CAG caused comprehensive metabolic alterations including the TCA cycle, glycolysis, membrane metabolism and catabolism, gut microbiota-related metabolism. On the other hand, electro-acupuncture treatment was found able to normalize a number of CAG-induced metabolomics changes by alleviating membrane catabolism, restoring function of neurotransmitter in brain and partially reverse the CAG-induced perturbation in gut microbiota metabolism. These findings provided new insights into the biochemistry of CAG and mechanism of the therapeutic effect of electro-acupuncture stimulations.

Chronic atrophic gastritis (CAG) is a functional gastrointestinal disease, characterized by atrophic gastric mucosa, reduced gastric acid secretion and pathological changeable epithelium along with intestine epithelium metaplasia^1^. CAG is often considered as precancerous lesions of gastric carcinoma^2^ which is ranked fourth in the cancer incidence and is currently the second most common cause of cancer-related deaths worldwide^3^. The incidence of CAG arises from repeated lesions of gastric superficial mucosa induced by various endogenous and exogenous factors such as emotional stress, physicochemical factors and microbial infection^4^. The common therapies of CAG focus on symptomatic treatment, which are often accompanied by unwanted side effects, incomplete remedy and recurrent episodes^5^.

Acupuncture has been used as an alternative therapy to CAG^6^. The acupuncture treatment is normally conducted by stimulating specific points in human body (i.e. acupoints) with fine needles^7^. Electro-acupuncture is a modification of conventional acupuncture practice, in which electro-acupuncture stimulates acupoints with an electrical current instead of manual manipulations. Multiple studies have shown that electro-acupuncture produces more consistent and reproducible results in both clinical and research settings^8^. In clinical trials, stimulation to acupoints of the stomach meridian such as *Sibai* (ST 2), *Liangmen* (ST 21), and *Zusanli* (ST 36) was found to be effective in treating various gastrointestinal diseases including functional dyspepsia^9^, irritable bowel syndrome^10^ and diarrhea^11^. This electro-acupuncture treatment was found to enhance the regularity of gastric myoelectrical activities, accelerate gastric emptying through the vagal pathway, regulate the content of gastrin, substance P (SP), epidermal growth factor (EGF), and transforming growth factor-α (TGF-α) in serum and gastric mucosa^12^. However, the mechanistic aspects of electro-acupuncture remain unknown. Since CAG pathological processes perturb the dynamic equilibrium of metabolism, metabolic differences are expected to be associated with the disease development. Therefore, establishment of CAG-related metabolic profiles is a potential approach to investigate the molecular mechanism of CAG and the beneficial effect of electro-acupuncture treatment on CAG.

As a systemic approach, metabolomics reflects the perturbed function of organisms based on metabolic changes caused by interventions in a holistic context^13^. This property is consistent with the holistic thinking of traditional Chinese medicine (TCM) suggesting that metabolomics has the potential to facilitate the understanding of the theory behind evidence-based Chinese medicine^14^. In the present study, the biochemical differences in biofluids (urine and serum) and tissues extracts (stomach, cortex and medulla) from CAG model and the metabolic impact of electro-acupuncture treatment were both investigated by a combination of high resolution ^1^H NMR spectroscopy and multivariate statistical analysis. Our results showed significant metabolic changes induced by CAG pathology. In addition, electro-acupuncture was found able to restore the CAG-induced metabolic perturbation back to normality.

## Results and Discussion

### Histological Morphology Examinations

**Figure 1:**
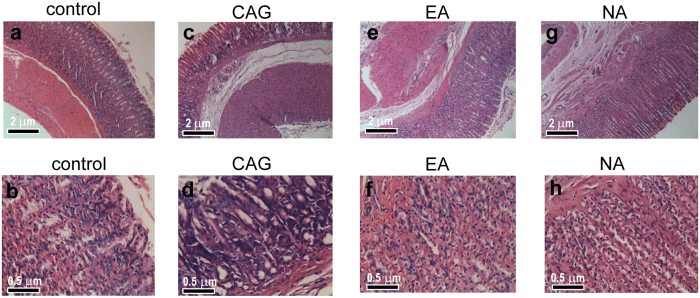
Photomicrographs of representative sections of gastric mucosa from four studied groups. The tissue sections were stained with hematoxylin-eosin. Scale bars represent 2 μm for the top row and 0.5 μm for the bottom row.

Microscopic examinations were conducted on the gastric mucosal tissues from the control, CAG model, EA and NA groups (Fig. 1). The control rats preserved the integrity of gastric mucosal enterocyte structure with well-arranged cells. The submucosa and muscularis were continuous without inflammatory cellular infiltration (Fig. 1a and b). On the other hand, thinning gastric mucosa layer and reduced gland cells were observed for the CAG rat. This is accompanied with incomplete glandular tube. In addition, significant cellular infiltration was visible in lymphocyte, plasmocyte and monocyte where cells were arrayed in disordered state with enlarged, variably sized, irregular nuclei and prominent nucleoli. Several mitoses can be observed in Fig. 1c and d. These observations illustrate a successful modelling of CAG rat. The electro-acupuncture treatment on the *Sibai, Liangmen*, and *Zusanli* acupoints of CAG rats rendered the gastric mucosal folds and thickness of gastric mucosa (Fig. 1e and f). A rather complete glandular tube structure was distinct with neat arranged cells and uniform nuclei with no pathologic mitosis. Based on the histopathological observations, electro-acupuncture stimulation on the *Sibai, Liangmen*, and *Zusanli* acupoints located on the Stomach meridian is considered as a significant curative treatment for the CAG animals. On the other hand, electro-acupuncture treatment on nonacupoints (the NA group) showed a thickened lamina propria mucosae, submucosa and muscularis with the proliferation of fibrous tissue. A histological observation showed incomplete mucosa gland morphology, variably sized nuclei and irregular cell alignment (Fig. 1g and h).

### Cellular Proliferation Examinations by ELISA and RT-PCR

It is known that CAG is closely related to gastric carcinoma^15^. The abnormality of cellular proliferation and apoptosis mechanism plays an important role in the course of malignant changing of gastric mucosa. The cellular over proliferation and abnormal apoptosis of gastric mucosa would probably induce gastric mucosa dysplasia and intestinal metaplasia. The regulation of several intracellular signaling pathways is believed to correlate with the over proliferation process where epidermal growth factor receptor (EGFR) is one of the critical pathways. EGFR is a member of the receptor tyrosine kinases (RTK) ErbB family^16^ and has been found to be overexpressed in various cancers, including non-small cell lung cancer^17^, colorectal cancer^18^, pancreatic cancer^19^, and gastric cancer^20^. High expression level of EGFR is associated with an increased risk of invasion or metastasis; while the inhibition of EGFR level leads to decreased cancer cell division, migration, angiogenesis and apoptosis in solid tumors.

It is known that activation of EGFR can subsequently activate Ras/Raf/MEK^21^ as well as its downstream protein kinase ERK1/2. Then, it turns to nucleus to regulate transcription factors such as c-myc, NF-κB, and promote the transcription of genes responsible for the process of cellular proliferation and apoptosis. Over proliferation of gastric mucosal cell was also accompanied by over-expression of PCNA^22^ that firmly tethers polymerases to DNA and increase their processivity for tens to thousands of nucleotides. The expression level of PCNA increases with degree of epithelial cell hyperplasia which reflects the state of cellular proliferation. Assessment of PCNA expression would be of great significance to investigate the clinical types of CAG and to diagnose the histological types of adenocarcinomas. Moreover, the growth and metastasis of gastric carcinoma is concerned with the co-regulation of multiple angiogenesis or anti-angiogenesis factors. VEGF is a potent angiogenic cytokine that induces a highly specific mitogen for endothelial cell and promotes the formation of neovascular. Pathogenesis of cancer has been reported to be directly related to VEGF level^23^. Ag-NORs are regulatory proteins for ribosomal DNA transcription, the levels of which present the transcription activity of rDNA (i.e. the activity level of cells).

**Figure 2:**
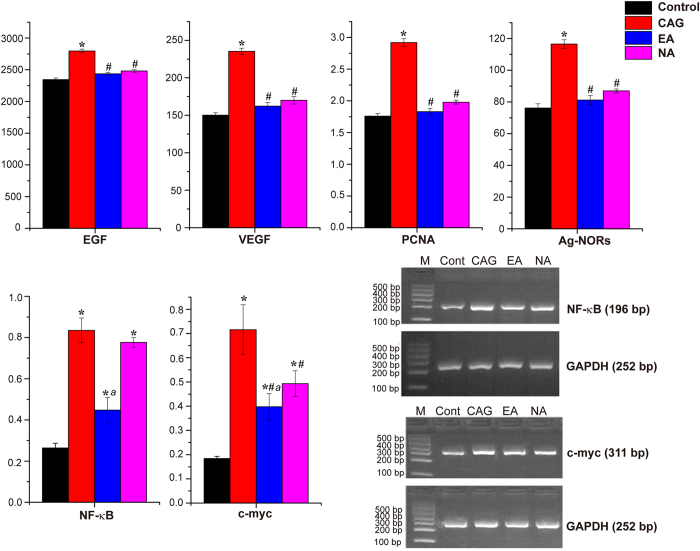
The expression of factors related to cellular proliferation in gastric mucosa and electrophoresis of NF-κB, c-myc mRNA and GAPDH mRNA RT-PCR product in gastric mucosal cells. *Indicate *p* < 0.05 statistical significance relative to the control group; ^#^indicate *p* < 0.05 statistical significance relative to the CAG group; ^a^indicate *p* < 0.05 statistical significance relative to the NA group.

In the current study, the expression level of PCNA, Ag-NORs, EGF and VEGF in gastric mucosal cells of CAG rats were determined by ELISA methods. As shown in Fig. 2, all of these four factors were markedly upregulated (*p* < 0.05) in the CAG rats, as compared with the controls. These results suggested enhanced expression of cell proliferation related factors following CAG modelling. In the EA group, significant decreases in the expression of PCNA, Ag-NORs, EGF and VEGF were observed compared to the CAG group, which indicated reduced expression of cell proliferation factor and further restraining the trends to malignant proliferation of gastric mucosal cells. Surprisingly, the NA group also exhibit lower expression of the four factors, although the regulation effect of electro-acupuncture on the *Sibai, Liangmen*, and *Zusanli* acupoints was stronger than that on nonacupoints in inhibiting the expression of the four factors.

The interesting finding may be explained by two aspects. Firstly, the curative effect of electro-acupuncture is a combined effect of both needling action as well as stimulation at specific acupoints. Although the non-acupoints lie away from the stomach meridian, needling stimulations at these non-acupoints may still exert metabolic response in the animals. In fact, in our previous electro-acupuncture study, we also found that EA-stimulated metabolic changes can be separated into two groups, the first group is non-specific metabolic changes due to acupuncture treatment, and the second group is due to stimulation at specific acupoints^24^.

Secondly, the four factors (i.e. PCNA, Ag-NORs, EGF and VEGF) were selected to observe the cellular proliferation in gastric mucosa based to published literatures as their expressions are sensitive to CAG-induced gastric mucosal lesions. However, the expressions of these genes maybe perturbed not specifically either due to the stimulation at acupoints or non-acupoints. This is in contrast to NF-κB genes expression, which was reduced specifically in response to stimulation at *Sibai, Liangmen and Zusanli* acupoints.

The gene expressions of c-myc and NF-κB that related to cellular proliferation in gastric mucosa were shown in Fig. 2. Compared to the control group, the elevation of NF-κB gene expression in CAG and NA groups and c-myc gene expression in CAG, EA, NA groups might be the indicator of over proliferation in gastric mucosal cells. The current result showed that electro-acupuncture treatment on the *Sibai, Liangmen*, and *Zusanli* acupoints of CAG rats (EA group) is more effective in reducing both c-myc and NF-κB genes expression, as compared to the NA group. The results are consistent with the histological findings.

### ^1^H NMR Profiles of Biological Fluids and Tissues from Rats

**Figure 3:**
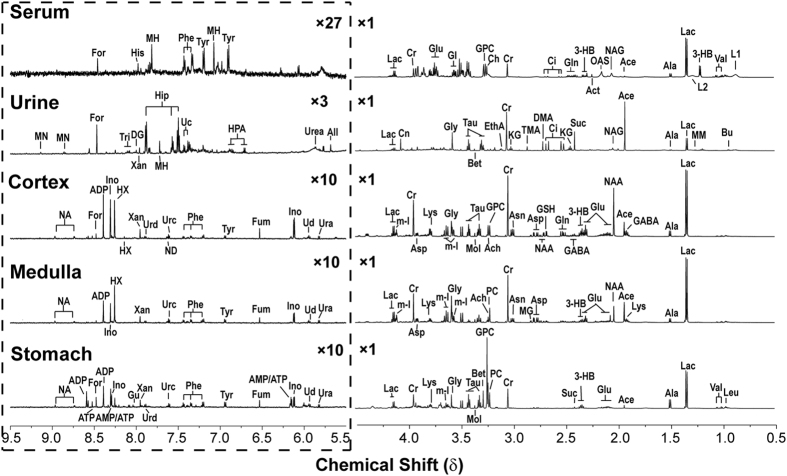
Typical ^1^H NMR spectra of serum, urine and aqueous cerebral cortex, medulla and stomach extracts from control rats. The region of δ 5.5–9.5 in the spectrum was magnified 27-, three- or tenfold in vertical expansion compared with the corresponding region of δ 0.5–4.5. (**Note:** *Ace, acetate; Ach, Acetylcholine; Act, acetone; ADP, Adenosine 3′,5′-diphosphate; Ala, alanine; All, allantoin; AMP, adenosine monophosphate; Asn, asparagine; Asp, aspartate; ATP, adenosine triphosphate; Bet, betaine; Bu, butyrate; Ch, choline; Ci, citrate; Cn, creatinine; Cr, creatine; DG, deoxyguanosine; DMA, dimethylamine; DMG, N,N-dimethylglycine; EthA, ethanolamine; For, formate; Fum, fumarate; Gl, glycerol; GABA, gamma-aminobutyrate; Gln, glutamine; Glu, glutamate; Gly, glycine; GPC, glycerophosphocholine; GSH, glutathione; Gu, guanosine; 3-HB, 3-hydroxybutyrate; His, histidine; HPA, hydroxyphenylacetate; HX, hypoxanthine; m-I, myo-inositol; Ino, inosine; KG, alpha-ketoglutarate; L1, LDL, CH3-*(*CH2) n-; L2, VLDL, CH3-*(*CH2) n-; Lac, lactate; Leu, leucine; Lys, lysine; MH, 1-methylhistidine; MM, methylmalonate; MN, 1-methylnicotinamide; Mol, methanol; NA, nicotinamide; NAA, N-acetylaspartate; NAG, N-acetylglutamate; OAS, O-acetylglycoprotein signals; Pan, pantothenate; PC, phosphocholine; Phe, phenylalanine; Suc, succinate; Tau, taurine; Tri, trigonelline; Tyr, tyrosine; Uc, urocanate; Ud, uridine; Ura, uracil; Val, valine; Xan, xanthine.*)

Representative ^1^H NMR spectra of biological fluids (serum and urine) and tissue extracts (cerebral cortex, stomach and medulla tissue extract) are shown in Fig. 3. The main peaks in the spectra were assigned to individual metabolites by a public NMR database (Human Metabolome Database V3.0, www.hmdb.ca) as well as an in-house developed NMR database.

**Figure 4:**
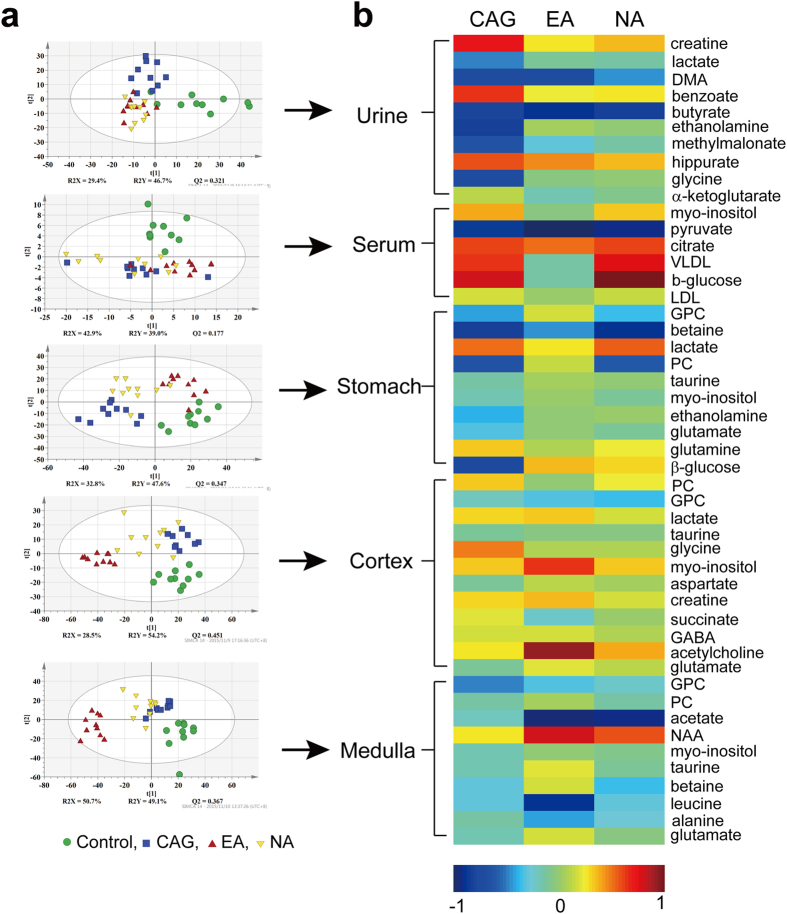
(**a**) PLS-DA scores plots derived from ^1^H NMR data of biofluid samples (urine and serum) and tissue samples (extracted from stomach, cortex and medulla). (**b**) Heat-map for metabolite changes color-scaled with the contents of metabolites. The warm color denotes an increase of metabolite levels whereas cold color indicates a decrease in the treated rats with respect to the control group.

The NMR datasets were analyzed using supervised PLS-DA method. The PLS-DA scores plots were shown in Fig. 4a. The results of corresponding permutation tests (data not shown) suggested the models were robust. The PLS-DA scores plot of data from blood serum, urine, and tissue samples (cortex, medulla and stomach) showed group separation for the four groups.

For all sample types, the controls are well separated from the CAG, EA, and NA groups. These results showed significant metabolic perturbations induced by CAG modelling. The effect of CAG modeling is the main group-separating factor observed in blood serum (second PLS-DA component) and urine (first PLS-DA component) samples. On the other hand, the PLS-DA scores plot of the stomach tissue extract showed that electro-acupuncture treatment on the *Sibai, Liangmen*, and *Zusanli* acupoints (i.e. the EA groups) normalized the metabolic perturbation caused by the CAG modeling. In the scores plot, the controls and EA groups are seen on the right side of the plot, while both the CAG and NA groups on the left. The result suggested that the improvement of CAG caused by electro-acupuncture treatment is observable in the stomach metabolic profile (Fig. 4b).

Notably, the effect of electro-acupuncture treatment contributed to the major metabolic variation in cortex and medulla (first PLS-DA component). The cortex metabolic profiles showed that the effect of non-specific electro-acupuncture treatment on both EA and NA groups. In contrast, the electro-acupuncture treatment on the specific *Sibai, Liangmen*, and *Zusanli* acupoints caused group separation between the EA group and three other groups (controls, CAG and NA groups). The finding may provide insight into the mechanism of electro-acupuncture treatment on CAG.

A heat-map of metabolic profiles from biofluids (urine and serum) and tissues (stomach, cortex and medulla) was shown in Fig. 4b to assess metabolic changes during the development of CAG and electro-acupuncture treated on *Sibai, Liangmen*, and *Zusanli* acupoints or non-acupoints. Metabolites responsible for group discrimination were picked out with their integrals of NMR peaks normalizing to corresponding buckets from control group. The logarithm of the ratio were reflected in the heat-map that the warm color (e.g. red) indicated the content of metabolite was higher relative to the control group, and cold color (e.g. blue) indicated a lower concentration as compared with the controls.

### Identification of Metabolic Alterations in Blood serum

**Figure 5:**
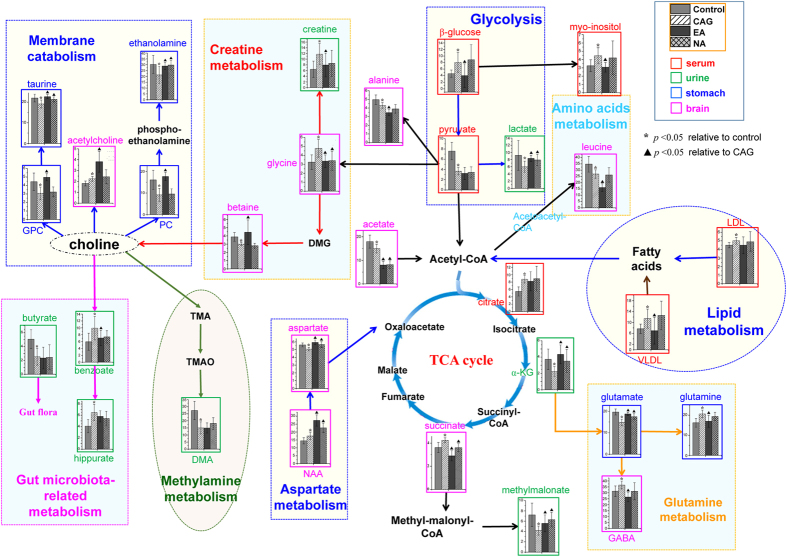
Summary of metabolic alterations due to CAG modeling and electro-acupuncture treatment in rat urine, serum, stomach and brain. The perturbed metabolic pathways and related participants are denoted by different colors.

Next, metabolic alterations caused by the CAG and the possible therapeutic effects of acupoint stimulations were analyzed in details. Systematic changes in multiple metabolic pathways (Fig. 5) were found to be disturbed by CAG modelling, which could be regulated by acupoints stimulations. Histograms of characteristic metabolites identified from biofluids and tissues were embedded into pathway graph to display the trends of changes.

In blood serum samples, the loadings of two-group PLS-DA models were displayed in the form of histogram with *p* values indicating the statistical significance relative to the control or CAG groups. The levels of myo-inositol, pyruvate, citrate, VLDL (very low-density lipoprotein, CH_3_-(CH_2_)_*n*_-), β-glucose and LDL (low-density lipoprotein, CH_3_-(CH_2_)_*n*_*-*) (all *p* < 0.05) were changed after CAG modelling, while myo-inositol, VLDL and β-glucose were found partially reversed upon electro-acupuncture treatment (all *p* < 0.05).

The elevation of serum lipid levels were correlated with enhanced EGF expression in CAG rats shown in Fig. 2. EGFR has been verified to play a regulatory role in hepatocyte proliferation and plasma lipid metabolism in adult male mice^25^. The livers of *Dsk5* mice, an animal model with gain of function in EGFR, exhibited decreased hepatic LDL receptor (LDLR) expression as well as fatty acid synthase (FAS) expression, providing metabolic mechanism for the elevated plasma LDL. Berrahmoune *et al*.^26^ reported a positive correlation between circulating EGF level and LDL levels. The data indicated that the cellular over-proliferation in gastric mucosa during the progression of CAG increased the activation of the EGFR signaling pathways, leading to upregulation the lipid metabolism. Electro-acupuncture treatment was found to inhibit EGF activation (Fig. 2), and decrease the plasma VLDL level with subtle reduction in LDL level.

Myo-inositol is a glucose derivative that may indicate the utilization of fat stores. Here in this study, CAG modeling caused an increase in myo-inositol, but this can be normalized following electro-acupuncture treatment. Together with increased serum β-glucose in CAG animals, as the results indicated abnormal energy metabolism in CAG pathological condition.

### Identification of Metabolic Alterations in Urine

Analysis of urinary metabolites may provide a comprehensive metabolome information response to physiological and pathological situations. Disease-dependent clustering of the control and CAG groups as well as treatment-dependent clustering of the CAG and EA groups were visible on PLS-DA score plot (Fig. 4a), and both models were validated to be robust by cross validation and random permutation tests. The changes in urinary metabolite concentrations upon CAG pathology mainly lay in creatine, lactate, DMA, benzoate, ethanolamine, methylmalonate, hippurate, glycine, α-ketoglutarate and butyrate (all *p* < 0.05), while most of the metabolites were recovered towards the normal levels upon electro-acupuncture treatment except DMA, butyrate (all *p* < 0.05).

As compared to the controls, increased concentration of hippurate, benzoate and decrease glycine level were observed in the urine of the CAG rats (both *p* < 0.05). This is in agreement with our previous report from acute gastric mucosal lesion (GML)^24^. Benzoate, by the conjugation of glycine, is converted to hippurate in mitochondria and is presumably catalyzed by benzoyl-CoA synthetase and benzoyl-CoA/glycine N-acyltransferase^27^. The abnormalities of urinary benzoate, hippurate and glycine suggested microflora dysbiosis attribute to destruction of gastric mucosa in CAG and GML, which might affect the absorption of nutrients. Electro-acupuncture treatment could effectively improve gastric mucosal morphology, preserve enterocyte microstructure (Fig. 1) and promote functional recovery. This could ultimately lead to partially improve the hippurate metabolic pathway in mitochondria.

DMA is a product of gut bacterial metabolism of dietary choline, although it also originated from the N-methylation of methylamines derived from glycine and sarcosine, methionine and the breakdown of creatine^28^. The concentration of creatine in urine is influenced by ingestion of animal protein. The digestive disturbances of body related to CAG course is believed to induce elevated creatine excretion into urine and further influence DMA concentration.

Methylmalonate plays an important role in maintaining the production of chemical energy through the TCA cycle^29^. Alpha-ketoglutarate (α-KG), also known as oxo-glutarate is a key intermediate in TCA cycle which combines with ammonia to form glutamate and then glutamine. The downregulation of α-KG and methylmalonate in urine were highly related to energy metabolism.

Ethanolamine is a substrate for the synthesis of phosphatidyl ethanolamine (PE) via CDP-ethanolamine, where PE account for over 60% of all phospholipids identified in human gastric mucosa. Lower PE were observed in CAG patients compared to healthy volunteers^30^, attributing to typical cellular rarefaction and a quantity of inflammatory infiltrate of CAG. The downregulation of PE synthesis route would probably result in a reduction of ethanolamine utilization and then lower excreted to urine. It is clear that electro-acupuncture treatment can render the structure of gastric mucosa and glandular tube and ameliorate infiltration of inflammation cells from the aspects of histological morphology. Therefore the level of ethanolamine could be increased in the EA group.

A significant decrease of urinary lactate level can be viewed as a result of changes in microbiota composition, especially an overgrowth of lactate-producing bacteria after CAG modeling^31^. Butyrate, a kind of short-chain fatty acids (SCFAs) synthesized by colonic microbiota, may induce apoptosis, cell cycle arrest and differentiation in human being^32^. The metabolic abnormality of butyrate in urine can be considered as an indicator that CAG pathology may also have negative effect on the microbiome of digestive system.

### Identification of Metabolic Alterations in Extracted Stomach Tissues

As identified by the histopathological results (Fig. 1), stomach was confirmed as a CAG-targeted organ. The metabolic changes induced by CAG and EA treatment in model rats were characterized by histogram where a set of endogenous metabolites existed in stomach have found to change in concentration upon CAG pathology (Fig. 5). It was found that changes in GPC, betaine, lactate, phosphocholine (PC), taurine, myo-inositol, ethanolamine, glutamate, formate and β-glucose (all *p* < 0.05) can be directly associated with CAG. The concentration of all these metabolites were reversed to different extent back towards the control state in response to EA stimuli on *Sibai, Liangmen*, and *Zusanli* acupoints indicating the effectiveness of electro-acupuncture therapy.

Betaine serves as organ osmolytes in biological systems. It plays a physiological role in endogenous mucosal protection. Decreased betaine concentration (*p* < 0.05) found in CAG rats indicated a dysfunction of gastric mucosal due to disease modeling. This result is consistent with our previous study on rats with acute gastric mucosal lesion^24^. Taurine has many diverse biological functions such as bile acid conjugation, antioxidation, osmoregulation, membrane stabilization and modulation of calcium signaling^33^. Taurine can improve drug induced-gastric damage by its antioxidant and/or membrane-stabilizing effects^34^. Lower level of taurine in stomach of CAG rats may be explained by metabolic disorder induced cell membrane breakage.

GPC and PC, comprising the total choline, mainly contribute to membrane synthesis and catabolism (Fig. 5). GPC is produced from the breakdown of the membrane phospholipid phosphatidylcholine in a 2-step reaction mediated via phospholipase A and lysophospholipase, which concomitantly leads to the formation of free fatty acids^35^. There is evidence showing that the increased concentrations of both GPC and PC with malignancy are not only due to increased cell proliferation but also because of a direct oncogenic protein regulation^36^. The downregulation of GPC and PC level in the present study reflects cellular membrane lesion of stomach caused by progressive CAG.

In addition, the concentrations of both glutamate and glutamine were affected by CAG modelling. They are produced from α-KG with the conjunction of ammonia through TCA cycle (Fig. 5). Glutamate together with acetyl-CoA can form N-acetylglutamate by the enzyme N-acetylglutamate synthase. Moreover, glutamate serves as the most abundant fast excitatory neurotransmitter in the mammalian nervous system and is probably perturbed by electro-acupuncture treatment. This finding is agreement with our previous result from electro-acupuncture treated on acute gastric mucosal lesion^24^.

In addition to the metabolites mentioned above, decreased myo-inositol, β-glucose and ethanolamine and increased lactate were detected in stomach tissue of the CAG rats. These four metabolites were also changed in the same direction in other sample types (Fig. 4).

### Identification of Metabolic Alterations in Extracted Brain Tissues

Metabolic perturbations in cortex and medulla were observed in PLS-DA score plots (Fig. 4) that electro-acupuncture stimuli has a marked effect onto these two tissues. The interaction of the nervous system with the digestive system has been understood by the discovery of the enteric nervous system (ENS) in the middle of the nineteenth century^37^,^38^. Since then, remarkable progress has been made in comprehending the bidirectional crosstalk between the brain and the digestive system. Inflammation of the digestive system, chronic abdominal pain syndromes and psychosocial stressors have been associated with the alterations in brain-gut interactions^39^. There is reason to believe the pathology of CAG would lead to significant metabolic changes in brain. The efficacy of electro-acupuncture treatment on CAG has been shown in improving the structure of gastric mucosa (Fig. 1) and regulating the expression of factors related to cellular proliferation (Fig. 2). Although the mechanism of electro-acupuncture stimulation remain to be established, there is evidence that acupoints stimulation can stimulate releases of hormones and neurotransmitters in the central nervous system which in turn assists the regulation of human physiological processes^40^.

Here, we had examined the effect of CAG and electro-acupuncture on the metabolism of cortex and medulla using NMR spectroscopy. In cortex homogenates, phosphocholine, GPC, lactate, taurine, glycine, myo-inositol, aspartate, creatine, succinate, GABA, acetylcholine and glutamate (all *p* < 0.05) may be related to CAG modeling. While in medulla homogenates, the alterations of GPC, phosphocholine, acetate, NAA, myo-inositol, taurine, betaine, leucine, alanine and glutamate (all *p* < 0.05) can be associated with CAG.

In the CAG animals, glutamate was found to decrease in both cortex and medulla, and GABA increased significantly in cortex. These changes most likely are the result of a shift in the steady-state equilibrium of glutamine-glutamate-GABA metabolic cycle (Fig. 5). First, reduced glutamate might be the result of decreased de novo synthesis via TCA cycle, as α-ketoglutarate can be transferred into glutamate via glutamate syntheses^41^. Then a reduction in glutamate might contribute to the conversion of GABA. GABA, the primary inhibitory neurotransmitter, is generated from glutamate in brain by glutamic acid decarboxylase^42^. The changes of GABA may indicate an adaptive response to exposure of gastrointestinal dysfunction by CAG.

The altered concentrations in PC and GPC were highlighted in the PLS-DA of stomach tissue extract indicating cellular membrane lesion of stomach. Comparatively, the increase of PC in brain tissue might be originated from the breakdown products of phosphatidylcholine (Fig. 5). Phosphatidylcholine is a major membrane constituent, thus perturbation in cell membrane metabolism in brain of CAG rats may warrant further study. Myo-inositol, a significant intracellular osmolyte is required by cells for synthesis of membrane phosphoinositides and for the maintenance of intracellular free myo-inositol levels. The reduced myo-inositol level in brain may reflect alterations in tissue osmolarity caused by CAG.

We also found a decrease of taurine in the two brain regions of CAG rats. It is known that taurine, a ubiquitous sulfur-containing β-amino acid, exhibits neuroprotective effect through stabilizing the cell membrane and modulating cellular calcium homeostasis and antioxidation^43^,^44^. Therefore the decline of taurine in this study may suggest its susceptibility to CAG pathology. Consistent with the discussion on stomach tissue, CAG modeling caused a decrease concentration of betaine in brain. Betaine helps to osmoregulate the extracellular osmolality and the changes in brain seem to be related to the osmolar imbalance in response to disease.

N-acetylaspartate (NAA) is synthesized in the mitochondria from aspartate and acetyl-coenzyme A (Fig. 5). It is a marker of neuronal functionality which found to increase in CAG rats as compared with controls. NAA is localized within neurons, and involved in synaptic process and can be considered a neuronal and axonal marker^45^. The elevated NAA concentration in brain could point to an enhanced or disturbed neuronal activity. Another neurotransmitter, acetylcholine functions as a neuromodulator in the brain, it alters the way of brain structures process information rather than transmit information. It is synthesized in neurons by the enzyme choline acetyltransferase from the compounds choline and acetyl-CoA (Fig. 5). Next, the enzyme acetylcholinesterase converts acetylcholine into choline and acetate. The increased acetylcholine and decreased acetate detected in brain were probably connected to perturbed neurotransmission process.

Creatine is a high energy compound necessary for the transport of phosphate groups and for maintaining appropriate cellular ATP levels. Therefore the increase ATP in brain of the CAG rats might reflect the alteration in brain energy metabolism. Lactate is an end product of anaerobic cellular metabolism, and increase in lactate is seen in pathologies that involve anaerobic conditions. Elevation of lactate levels may suggest an enhanced anaerobic metabolism and an oxidative injury effect related to impaired pyruvate oxidation in brain. Alanine is one of the most abundant amino acids in the body, which can be synthesized from pyruvate and branched chain amino acids such as leucine. It is extensively used as an intermediate in many metabolic pathways such as glycolysis, gluconeogenesis, TCA cycle and alanine cycle. The alteration of alanine and leucine in brain could be seen as another evidence for abnormal energy metabolism caused by CAG.

From the histograms of each metabolite (Fig. 5), metabolic concentrations were compared distinctly between the control, CAG, EA and NA groups. Metabolic cycle related to neuro conduction, glutamine-glutamate-GABA cycle was adjusted by electro-acupuncture stimulation while other neurotransmitters as NAA, acetate and acetylcholine were less affected. Limited observations of the regulating action in central nervous system were attributed to the static measurement of NMR spectra at single time points. It is believed that real-time dynamic information about neural signaling can provide further insight into the effect of electro-acupunture on brain metabolism. Besides, metabolites that play roles in the integrity of cellular membrane and tissue osmolarity as PC, GPC, myo-inositol, taurine and betaine were also regulated by electro-acupuncture treatment while metabolites related to brain energy metabolism such as creatine, lactate, alanine and leucine showed no significant improvement.

## Conclusion

In the current study, an integrated NMR analysis of multiple biological samples along with pathological examination and gene expression and protein assessment were employed to investigate the metabolic alterations due of CAG and curative effects of electro-acupuncture on this disease. Metabolomics analysis of serum, urine, stomach, cortex and medulla samples revealed the perturbation in multiple metabolic pathways, including membrane metabolism, amino acids metabolism, gut microbiota-related metabolism and energy metabolism in CAG animals. On the other hand, electro-acupuncture stimulations showed beneficial effects by renormalizing many CAG-induced metabolic changes. Notably, our current study also showed that electro acupuncture at non-acupoints also induced changes in expressions of selected genes. The current findings suggested that acupuncture studies should include both controls (with no acupuncture treatment) as well as controls with treatment at non-acupoints to distinguish the non acupoint-specific changes and acupoint-specific changes. Recommendations for future studies also include consecutive observation at different stages of CAG progression and a longer term electro-acupuncture interventions. In addition, the current work also showed the potential of metabolomic applications in revealing biological mechanism of traditional Chinese medicine practices such as electro-acupuncture.

## Experimental Procedures

### Ethical Statement

Animal care and experimental procedures used in the current study were approved by the Animal Care and Use Committee of Xiamen University (Permit Number: SCXK 2008-0001). The study was carried out adhering to guidelines provided by National Institutes of Health for the Care and Use of Laboratory Animals and all efforts were made to minimize suffering of animals.

### Animal Handling

All 40 healthy male rats (150 ± 20 g weight) used in this study were housed individually in metabolism cages. The animal room was under controlled condition (temperature, humidity, and a 12-hour light-dark cycle) and the rats were provided with food and water *ad libitum.*

After one week’s adaptation, the rats were randomly separated into four groups (n = 10 per group): (1) control group, (2) chronic atrophic gastritis (CAG) model group, (3) CAG rats with electro-acupuncture treatment on the stomach meridian (SM) acupoints (EA group), and (4) CAG rats with electro-acupuncture on non-acupoints (NA group). The control group were freely given clean water and fed with standard rat diet. The CAG rat models were induced by irregular fasting, compulsive sporting^1^, and by mixing 2% sodium salicylate and 30% alcohol and poured down the throats of the CAG rats for 12 weeks to stimulate gastric mucosa in these animals.

### Electro-acupuncture Treatment

After CAG modeling, both EA and NA groups were treated with electro-acupuncture (a 30-minute session per day) for two weeks. For the EA group, three functional acupoints located in the Stomach meridian including *Sibai* (ST 2), *Liangmen* (ST 21), and *Zusanli* (ST 36) were treated with electro-acupuncture. For the NA group, three non-acupoints were selected for treatment. The non-acupoints are located 5 mm above each of *Sibai, Liangmen*, and *Zusanli* acupoints^46^. It has been a general consensus to use locations at 5 mm away from acupoints (e.g. *Sibai, Liangmen and Zusanli* acupoints in the current study) as non-acupoints in animal experiments. These non-acupoints do not lie on the stomach meridian and do not overlap with other known acupoints.

The locations of *Sibai* (ST 2), *Liangmen* (ST 21), and *Zusanli* (ST 36) acupoints were determined according to Government Channel and Points Standard GB12346-90 of China and “The Veterinary Acupuncture of China”. Two-channel electrical stimulations were performed with a pulse generator (Model G6805-2; Qingdao Xinsheng Medical Instrument Factory, Shandong, China) via stainless-steel acupuncture needles of 0.25 mm in diameter. The electrical stimuli consisted of intermittent and irregular waves (intermittent wave: 4 Hz; irregular wave: 50 Hz) with voltage ranging from 2 to 4 V. The electrical intensity was just strong enough to elicit slight twitches of the hind limbs.

### Histopathology

The randomly selected samples of the gastric mucosa samples from rats were taken and placed in the phosphate-buffered 10% formalin for histological assessment. After sample dehydration, the biopsies embedded in wax were sectioned at 5 μm and stained with hematoxylin and eosin for histopathological examination by light microscopy.

### Enzyme linked immunosorbent assay (ELISA) assessment

After electro-acupuncture treatment, all rats were sacrificed by exsanguination and the gastric glands about 1 cm × 1 cm were removed and weighed. Each tissue sample was cut into pieces and mixed with 0.1 M ice-cooled phosphate buffered saline (PBS) by a proportion of 1:9 (w/v). This mixture were homogenated on ice at 10 000 rpm and then transferred to 2 mL tube to centrifuge for 15 min (3000 rpm, 4 °C). The upper supernatants were transferred into clean tubes and stored at −80 °C until used.

The proliferating cell nuclear antigen (PCNA), Ag-nucleolar organization regions (Ag-NORs), epidermal growth factor (EGF), vascular endothelial growth factor (VEGF) level of the above samples were determined using an ELISA kit purchased from BD Biosciences (San Jose, California, USA). The ELISA-based method was carried out according to the protocol provided by the manufacturer.

### Real time fluorescent quantitative RT-PCR

Total RNA of gastric mucosa tissue was extracted by Trizol reagent according to the protocol provided by the manufacturer (Beijing Dingguo Changsheng Bioscience Ltd.). Total RNA was treated with DNase Ι to eliminate genomic DNA contamination, followed by synthesis of the first strand using the reverse transcription system. Real-time PCR was performed in 25 μL of reaction solution containing 12.5 μL 2 × iQSYBR Green Supermix (Bio-Rad Laboratories, Hercules, California, USA), 300 nM primers and complementary DNA. The cycles for PCR were as follows: 95 °C for 7 min, 40 cycles of 95 °C for 20 s, 54 °C for 30 s and 72 °C for 30 s. Melting curves were determined by heat denaturing PCR products over a 35 °C temperature gradient at 0.2 °C/s from 60 to 95 °C. Fold changes in the messenger RNA (mRNA) levels of target genes related to their variant control glyceraldehyde-3-phosphate dehydrogenase (GAPDH) (146 kDa) were calculated as described^47^. The primer sequences are shown in Table 1. Then the PCR products were analyzed by 1.5% agarose gel electrophoresis, ethidium bromide staining for 30 min and photographed under UV transilluminator. Signals were quantified by density analysis of the digital images using Quantity One ver. 4.52 (Bio-Rad Laboratories, Hercules, California, USA) and quotients of NF-κB, c-myc to internal reference GAPHD were calculated respectively to indicate the relative expression.

### Biological sample collection and NMR experiments

After the fourteen-day electro-acupuncture treatment course, 24 hours’ urine samples of each rats were collected in ice-cooled vessels containing 1% sodium azide (0.1 mL) and immediately frozen at −80 °C. Animals were sacrificed by exsanguination under isoflurane anesthesia. Whole blood was drawn from carotid arteries using a catheter and left to clot at room temperature for 1 h. Then, the samples were centrifuged at 10 000 rpm for 10 min at 4 °C to remove particulate contaminants and then stored at −80 °C for NMR experiments. The cerebral cortex, stomach, and medulla tissue samples were excised and snap-frozen in liquid nitrogen immediately for tissue extraction. These samples were stored at −80 °C until used.

For urine samples, a volume of 300 μL was mixed with 300 μL phosphate buffer solution (1.5 M K_2_HPO_4_/NaH_2_PO_4_, pH 7.4, 99.9% D_2_O) to reduce pH variation across samples. 0.3 mM TSP (3-(trimethylsilyl) propionic-2,2,3,3-d4 acid sodium salt) was used as an internal reference standard at δ 0.0. The mixture was left to stand for 10 min and centrifuged at 10 000 rpm at 4 °C for 10 min to remove precipitates. Then, 500 μL of the supernatants were transferred into a 5 mm NMR tube and analyzed by NMR spectroscopy. ^1^H NMR spectra of these samples were acquired using the standard NOESYPR1D pulse sequence using a 600 MHz Bruker spectrometer at 296 K. For each sample, 64 free induction decays (FIDs) were collected into 40 K data points over a spectral width of 12 000 Hz with a relaxation delay of 0.23 ms.

For blood serum samples, a volume of 400 μL blood serum was mixed with 200 μL phosphate buffer solution (90 mM K_2_HPO_4_/NaH_2_PO_4_, pH 7.4, 99.9% D_2_O). Then the mixture was transferred to centrifugal tube and centrifuged at 10 000 rpm for 10 min at 4 °C. 500 μL of supernatant was piped into 5 mm NMR tube for NMR experiment. ^1^H NMR spectra of these samples were acquired using a 600 MHz Bruker spectrometer at 296 K. Standard 1D ^1^H spectra were acquired with a water-suppressed CPMG pulse sequence. For each sample, 64 FIDs were collected into 20 K data points over a spectral width of 12 000 Hz with a relaxation delay of 0.23 ms.

The polar metabolites in rat tissue were extracted according to the protocol established by Wu *et al*.^48^. Briefly, pre-weighed cerebral cortex, stomach, medulla samples (300 mg per sample) were homogenated in 1.4 mL of CH_3_OH and 0.56 mL of H_2_O and then vortexed for 60 s. After 10 min partitioning on ice, the samples were centrifuged for 5 min (10 000 rpm, 4 °C). The upper supernatants were transferred into 2 mL tubes, and lyophilized to remove CH_3_OH and H_2_O. The extracts were reconstituted into 500 μL D_2_O containing 1 mM TSP, then transferred into 5 mm NMR tubes and analyzed by NMR spectroscopy. ^1^H NMR spectra of these samples were acquired using a Bruker 600 MHz spectrometer at 296 K. Standard 1D ^1^H spectra were acquired with a ‘ZGPR’ pulse sequence. For each sample, 64 FIDs were collected into 64 K data points over a spectral width of 12 000 Hz with a relaxation delay of 6.5 μs and an acquisition time of 2.66 s. For data analysis, metabolites in the biofluids and tissue ^1^H NMR spectra were assigned with reference to published data^49^, HMDB database (http://www.hmdb.ca/) and an in-house developed NMR database.

### Data Preprocessing and multivariate statistical analysis

The acquired ^1^H NMR spectra were phased and baseline corrected using MestReNova v9.0.1 software (Mestrelab Research S.L.). ^1^H NMR spectra were either referenced to the internal lactate CH_3_ resonance at 1.33 ppm (for serum spectra) or a single peak of TSP at 0.0 ppm (for urinary and tissue spectra). All spectra were also peak-aligned manually to overcome unwanted peak-shift problem^50^. Then the chemical shift ranges which include the resonances from water and urea, as well as the baseline (peak-free) regions were removed from further analysis. Then the spectra over the ranges of 0.5–9.0 ppm for blood serum samples and 0.5–10.0 ppm for urine and tissue samples were binned into buckets with fixed width of 0.002 ppm. Prior to statistical analysis, the bucketed data were normalized by the method of probabilistic quotient normalization^51^ to compensate for the differences in overall concentrations of the samples.

The NMR spectral data were saved as a Microsoft Excel format files and imported into SIMCA-P software (version 14, Umetrics AB, Umea, Sweden) for multivariate analysis. The partial least squares discriminant analysis (PLS-DA) was utilized to overview samples clustering responsible for CAG modelling and electro-acupuncture treatment. Then, the metabolic signatures that contribute to the clustering were determined. R^2^X and Q^2^ values were used to describe the quality of multivariate model, where R^2^X is defined as the proportion of variance in the data explained by the models and indicates goodness of fit, and Q^2^ is defined as the proportion of variance in the response data Y (*i.e.*, class attributes of the samples) predicted by the model and indicates predictability. In addition, two-group PLS-DA models were also constructed (two-group comparison between the control, CAG and EA groups) and the corresponding VIP values were used as parameters for variable selection. A variable with VIP value greater than one is considered important and is selected to be investigated for its statistical significance between groups by unpaired t-test. (OriginPro ver. 8.1). The histograms from selected variable were embedded into the pathway graph.

## Additional Information

**How to cite this article:** Xu, J. *et al*. NMR-based metabolomics Reveals Alterations of Electro-acupuncture Stimulations on Chronic Atrophic Gastritis Rats. *Sci. Rep.* **7**, 45580; doi: 10.1038/srep45580 (2017).

**Publisher's note:** Springer Nature remains neutral with regard to jurisdictional claims in published maps and institutional affiliations.

## Figures and Tables

**Table 1:** The target genes and corresponding primer sequences.

NF-κB	Forward primer:	5′-AATTTGGCTTCCTTTCTTGGCT-3′
	Reverse primer:	5′-CTGCGATACCTTAATGACAGCG-3′
c-myc	Forward primer:	5′-TCCAGCGAGAGACAGAG-3′
	Reverse primer:	5′-GCAGAGGCAGAGAACAC-3′
GAPDH	Forward primer:	5′-AGAAGGCTGGGGCTCATTTG-3
	Reverse primer:	5′-AGGGGCCATCCACAGTCTTC-3′
